# Evaluation of the Optimal Position for Vedolizumab in the Japanese Treatment Paradigm for Ulcerative Colitis Using Markov Modeling

**DOI:** 10.1093/crocol/otaa017

**Published:** 2020-03-18

**Authors:** Akihito Uda, Yuki Eto, Yuxin Li, Hiroyuki Matsuda, Sven Demiya, Tomoyuki Watanabe, Mihoko Ota, Ryuichi Iwakiri, Ataru Igarashi

**Affiliations:** 1 Japan Medical Office, Takeda Pharmaceutical Co. Ltd., Tokyo, Japan; 2 IQVIA Solutions UK Ltd., London, UK; 3 IQVIA Solutions Japan KK, Tokyo, Japan; 4 Unit of Public Health and Preventive Medicine, School of Medicine, Yokohama City University, Yokohama, Kanagawa, Japan; 5 Department of Health Economics and Outcomes Research, Graduate School of Pharmaceutical Sciences, The University of Tokyo, Bunkyo, Tokyo, Japan

**Keywords:** biologic therapy, Japan, Markov process, ulcerative colitis, vedolizumab

## Abstract

**Background:**

This analysis assessed the optimal position of vedolizumab for Japanese patients with ulcerative colitis.

**Methods:**

A Markov model was used to evaluate the performance of 4 treatment algorithms of vedolizumab position: after azathioprine (Algorithm 1); after tacrolimus/cytapheresis (Algorithm 2); after a first anti-tumor necrosis factor alpha (anti-TNFα) (Algorithm 3); and after a second anti-TNFα before colectomy (Algorithm 4).

**Results:**

Algorithm 1 was the dominant strategy, with an incremental benefit over the other algorithms of 0.028–0.031 quality-adjusted life years.

**Conclusions:**

This simulation predicts that introducing vedolizumab immediately after a thiopurine and before other therapies will provide most benefit.

## INTRODUCTION

Ulcerative colitis (UC) is a relapsing/remitting inflammatory bowel disease (IBD) of unknown etiology that is characterized by chronic inflammation of the colonic mucosa.^1, 2^ Across Asia (including Japan), the incidence of UC has been increasing in line with industrialization and urbanization; however, it is still much lower than in Western regions, such as Europe and North America.^3, 4^ Accordingly, the prevalence of UC in Japan has increased from 18.1 per 100,000 persons in 1991 to 121.9 per 100,000 persons in 2013.^4^ As of December 2014, approximately 170,000 patients with UC were receiving treatment in Japan.^5^

Considerable advances in the treatment of UC have been made over recent years, including the introduction of biologics, such as the tumor necrosis factor-alpha (TNFα) antagonists (adalimumab, infliximab, and golimumab), which have transformed the treatment of steroid-dependent disease.^6^ TNFα antagonists have demonstrated substantial effectiveness in this setting; however, 10–30% of patients with IBD do not respond to initial treatment with a TNFα antagonist and 23%–46% of those who do respond lose their response over time.^7^ Additionally, TNFα antagonists are associated with some safety considerations, such as an increased risk of potentially serious opportunistic infections.^6^

Vedolizumab represents a new type of biologic with a novel mode of action. It is a humanized monoclonal antibody that binds selectively to α _4_β _7_ integrin without inducing significant systemic immunosuppression and blocks lymphocyte infiltration to the gut tissue.^8^ Vedolizumab demonstrated efficacy vs placebo in patients with moderate-to-severely active UC (as both induction and maintenance therapy) in the pivotal GEMINI 1 trial,^9^ and is now widely approved in multiple countries/regions (including Japan) for the treatment of moderate-to-severe UC.^10–12^ Furthermore, a post-hoc analysis of the GEMINI 1 data showed that vedolizumab was more efficacious than placebo irrespective of prior TNFα antagonist exposure, although TNFα antagonist-naive patients achieved higher rates of response and remission than pretreated patients.^13^ The efficacy and safety of vedolizumab were also demonstrated in a phase 3 trial of Japanese patients with active UC; vedolizumab treatment was associated with numerically higher response rates compared with placebo when used as induction therapy; and was significantly superior to placebo in maintaining clinical remission when used as maintenance therapy.^14^

Current Japanese treatment guidelines (published before the approval of vedolizumab) recommend corticosteroids as a standard of care for more severely active UC, followed by step-up treatment with a thiopurine immunomodulator (azathioprine or 6-mercaptopurine [6-MP]; for maintenance but not induction of remission), tacrolimus or cyclosporine A, cytapharesis, or a TNFα antagonist.^1^ Surgery (colectomy) may also be considered for patients with intestinal perforation, massive bleeding, or toxic megacolon.^1^ Although these guidelines are a valuable tool for treatment decision-making, there is no standard treatment algorithm to follow (especially for patients with steroid-dependent UC), nor is there any certainty around where, in the treatment paradigm, new agents should be included. Mathematical simulation offers an alternative option to evaluate the optimal clinical position of new agents (with different mechanisms of action) within the treatment pathway. A United States (US) simulation modeling was used previously to assess the optimal position for vedolizumab in the treatment of UC through comparison of treatment algorithms for patients with moderate-to-severely active disease.^15^ The model predicted that treatment algorithms which positioned vedolizumab before other therapies should be considered for patients with steroid-dependent, moderate-to-severely active UC, supporting the use of vedolizumab as a steroid-sparing initial treatment in this subset of patients. In the current study, we utilize these previously reported simulation modeling techniques^15^ to predict where, in the treatment pathway, the use of vedolizumab might provide the optimal clinical benefit for Japanese patients with moderate-to-severely active, steroid-dependent UC.

## MATERIALS AND METHODS

### Ethical Considerations

This article is an original contribution that has not been published previously nor it is under consideration for publication elsewhere. All authors meet the authorship criteria put forth by the International Committee of Medical Journal Editors and have approved the final draft before submission. As the paper reports a retrospective health outcomes analysis, ethical permissions were not required.

### Model Construct

A Markov simulation model was developed to evaluate where, in the Japanese treatment paradigm, vedolizumab might provide the optimal benefit for patients with moderate-to-severely active, steroid-dependent UC. The model, which was based on the previous US design,^15^ was developed by IQVIA Solutions Japan KK (Tokyo, Japan), under supervision of the authors. It comprised a base-case scenario of a 40-year-old Japanese male with moderate-to-severely active, steroid-dependent UC. This base-case reflects a “typical” patient in Japan (by disease characteristics, mean age, and median [most common] sex) and was established by analyzing the records of 642 patients in the Medical Data Vision (MDV) administrative health claims database (https://www.mdv.co.jp) who were identified as having steroid-dependent UC. The analysis period was defined as July 1, 2017 to December 31, 2018. Patients with steroid-dependent UC were identified as individuals aged ≥18 years at the index date (ie, the date of first prescription of a newly prescribed treatment of interest [TNFα antagonist, tacrolimus, ciclosporin, cytapheresis, or ciclosporin plus azathioprine or 6-MP] within the identification period of January 1, 2018 to December 31, 2018) with ≥1 definitive diagnosis of UC (International Classification of Diseases 10th edition code: K51) during a 6-month look-back period (including the index date), and a first-line prescription of azathioprine or 6-MP before the index date.

The base-case analyses were run over a 1-year time horizon (ie, the typical length of a UC induction and maintenance study) using 3-month cycle lengths. A 3-month cycle was considered optimal to capture the frequency of key clinical events. However, a half-cycle correction for outcomes was applied to reduce the potential for bias in the estimates. In accordance with Japanese health technology assessment guidelines, a discount rate of 2% per annum was applied to all rewards in scenarios where the simulations were extended beyond 1 year.

**Figure 1.**
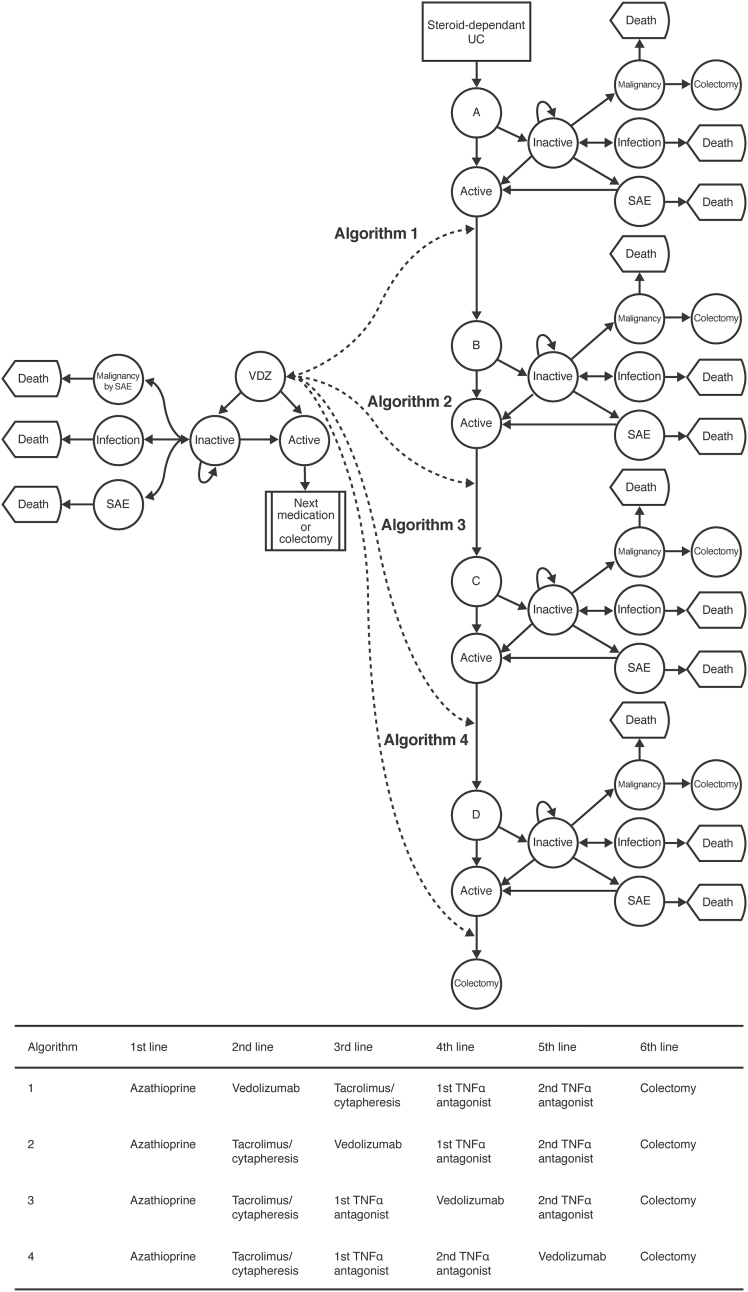
Overview of model structure and vedolizumab insertion sites. A = azathioprine; B = tacrolimus/cytapheresis OR first TNFα antagonist; C = first TNFα antagonist OR second TNFα antagonist; D = second TNFα antagonist OR colectomy. For each medication, individuals were exposed to the risk of not responding or entering remission, subsequently flaring, experiencing an SAE requiring drug discontinuation, developing a serious infection requiring a temporary hold in treatment, and developing lymphoma. It was presumed that colectomy would be the least preferred option for all patients. SAE, serious adverse event; TNFα, tumor necrosis factor alpha; UC, ulcerative colitis; VDZ, vedolizumab.

Four potential algorithms (all based on a step-up treatment paradigm that is consistent with current Japanese treatment guideline recommendations for steroid-dependent UC)^1^ were constructed and compared with each other (Fig. 1), each inserting vedolizumab into the algorithm at a separate position in the model. In all 4 algorithms, individuals intially received azathioprine as first-line therapy (initially as an add-on to steroid treatment, with tapered discontinuation of steroid therapy if remission was achieved). Further lines of therapy were, in order, tacrolimus/cytapheresis (in combination), a first TNFα antagonist, a second TNFα antagonist, and colectomy. It was presumed that colectomy would be the least preferred option for all patients. MDV administrative health claims database was analyzed to estimate the number of patients who would skip tacrolimus/cytapheresis and receive a TNFα antagonist after azathioprine therapy. Vedolizumab was incorporated in the simulations after azathioprine in Algorithm 1, after tacrolimus/cytapheresis in Algorithm 2, after the first TNFα antagonist in Algorithm 3, and after the second TNFα antagonist in Algorithm 4. For each medication, individuals were exposed to the risk of not responding or entering remission, subsequently flaring, experiencing a serious adverse event (SAE) requiring drug discontinuation, developing a serious infection requiring a temporary hold in treatment, and developing a malignancy (lymphoma).

In all cases, individuals first entered an induction phase in which they could achieve steroid-free remission, a clinical response without steroid-free remission, or no response. Individuals could also experience an SAE requiring drug discontinuation or a serious infection requiring a temporary treatment hold. SAEs and serious infections lasting longer than one 3-month cycle without resolution were only counted as a single event. Patients obtaining a clinical response or remission remained on the same treatment, and entered the maintenance phase; all other patients received the next treatment in the algorithm. During maintenance, individuals could experience a loss of response, an SAE requiring treatment discontinuation, a serious infection requiring a temporary hold in therapy, or malignancy. Individuals with an SAE requiring drug discontinuation, no response, or loss of response received the next therapy in the algorithm. At all stages, it was assumed that patients transition between health states at the beginning of each cycle and no more than one transition may occur per cycle. It was also assumed that patients may transition to death from any health state.

A one-cycle duration of increased peri-operative mortality, poorer quality of life, all-cause, age-specific mortality, and risk of a complicated postoperative disease course were presumed for patients who preceded to colectomy, based on a prior report.^16^ Individuals who underwent successful colectomy received a disutility linked with living with a J-pouch, as published previously.^17^

### Model Inputs

Base-case transition probabilities (clinical inputs), mortality/complication/malignancy rates, and quality-of-life utilities (preferences) to populate the Markov simulation model were identified through two systematic literature reviews: one conducted for this study (to identify Japanese-specific data; see Japanese Systematic Literature Review in Supplementary File 1) and one conducted previously (for US data; ^15^ used when Japanese data were not available). As the analysis was conducted from a Japanese public healthcare perspective, wherever possible, Japanese data were used to estimate the base-case parameters. Base-case estimates and all assumptions made relating to these values are summarized in Table 1.

**Table 1.** Transition Probabilities, Mortality/Complication/Malignancy Rates, and Utility Estimates

Description	Base-case Definition	Source
Azathioprine induction		
Probability of remission	0.2368	Panaccione et al, 2014^18^
Probability of clinical response	0.2632	Panaccione et al, 2014^18^
Probability of SAE	0.0886	Panaccione et al, 2014^18^
Probability of serious infection	0.0127	Panaccione et al, 2014^18^
Azathioprine maintenance		
Probability of flare while in remission over 1 year	0.8637	Timmer et al, 2012^19^
Probability of flare with clinical response over 1 year	0.8023	Scott et al, 2018^15^
Probability of SAE requiring discontinuation of maintenance over 1 year	0.0198	Timmer et al, 2012^19^
Probability of serious infection over 1 year	0.0032	Panaccione et al, 2014^18^
Tacrolimus/cytapheresis induction		
Probability of remission	0.4000	Yamamoto et al, 2016^20^ (for tacrolimus)
Probability of clinical response	0.6200	Yamamoto et al, 2016^20^ (for tacrolimus)
Probability of SAE	0	Yamamoto et al, 2016^20^ (for tacrolimus)
Probability of serious infection	0	Yamamoto et al, 2016^20^ (for tacrolimus)
Tacrolimus/cytapheresis maintenance		
Probability of flare while in remission over 1 year	0.8089	Yamamoto et al, 2016^20^; Yokoyama et al, 2014^21^ (for tacrolimus and cytapheresis [weighted]; assumed rate of cytapheresis = annual rate)
Probability of flare with clinical response over 1 year	0.8324	Yamamoto et al, 2016^20^ (for tacrolimus; assumed to be same as remission)
Probability of SAE requiring discontinuation of maintenance over 1 year	0.0100	Yamamoto et al, 2016^20^ (for tacrolimus)
Probability of serious infection over 1 year	0	Yamamoto et al, 2016^20^ (for tacrolimus)
TNFα antagonist induction (first/second use)		
Probability of remission	0.1970	Suzuki et al, 2015^24^; Hibi et al, 2017^23^ (ADA = weighted average between IFX/GOL)
Probability of clinical response	0.5223	Suzuki et al, 2015^24^; Suzuki et al, 2014^22^; Hibi et al, 2017^23^
Probability of SAE	0.0340	Suzuki et al, 2014^22^ (assumed IFX and GOL = ADA)
Probability of serious infection	0.0170	Suzuki et al, 2014^22^ (assumed IFX and GOL = ADA)
TNFα antagonist maintenance therapy (first/second use)		
Probability of maintaining remission up to 30 weeks	0.8659	Scott et al, 2018^15^
Probability of maintaining clinical response up to 30 weeks	0.7343	Suzuki et al, 2015^24^ (IFX input used for ADA and GOL)
Probability of maintaining remission after 30 weeks	0.8409	Hibi et al, 2017^23^ (assumed ADA and GOL = IFX)
Probability of maintaining clinical response after 30 weeks	0.7025	Suzuki et al, 2014^22^; Hibi et al, 2017^23^ (assumed ADA and IFX = GOL)
Probability of SAE requiring discontinuation of maintenance over 1 year	0.1350	Suzuki et al, 2015^24^; Suzuki et al, 2014^22^; Hibi et al, 2017^23^
Probability of serious infection over 1 year	0.0203	Suzuki et al, 2014^22^ (assumed IFX and GOL = ADA)
Vedolizumab induction (TNFα antagonist naïve)		
Probability of remission	0.2780	Motoya et al, 2018^14^ (assumed outcomes at week 10 = week 12)
Probability of clinical response	0.5320	Motoya et al, 2018^14^ (assumed outcomes at week 10 = week 12)
Probability of SAE	0.0610	Motoya et al, 2018^14^ (assumed outcomes at week 10 = week 12)
Probability of serious infection	0	Motoya et al, 2018^14^ (assumed no infections and outcomes at week 10 = week 12)
Vedolizumab induction (TNFα antagonist exposed)		
Probability of remission	0.0940	Motoya et al, 2018^14^ (assumed outcomes at week 10 = week 12)
Probability of clinical response	0.2710	Motoya et al, 2018^14^ (assumed outcomes at week 10 = week 12)
Probability of SAE	0.0610	Motoya et al, 2018^14^ (assumed same as TNFα antagonist naïve and outcomes at week 10 = week 12)
Probability of serious infection	0	Motoya et al, 2018^14^ (assumed no infections and outcomes at week 10 = week 12)
Vedolizumab maintenance (TNFα antagonist naïve)		
Probability of maintaining remission	0.8227	Motoya et al, 2018^14^ (assumed outcomes at week 60 = week 52)
Probability of maintaining clinical response	0.7596	Motoya et al, 2018^14^ (assumed outcomes at week 60 = week 52)
Probability of SAE requiring discontinuation of maintenance over 1 year	0.0060	Motoya et al, 2018^14^ (assumed outcomes at week 60 = week 52)
Probability of serious infection over 1 year	0	Motoya et al, 2018^14^ (assumed no infections and outcomes at week 60 = week 52)
Vedolizumab maintenance (TNFα antagonist exposed)		
Probability of maintaining remission	0.8012	Motoya et al, 2018^14^ (assumed outcomes at week 60 = week 52)
Probability of maintaining clinical response	0.7708	Motoya et al, 2018^14^ (assumed outcomes at week 60 = week 52)
Probability of SAE requiring discontinuation of maintenance over 1 year	0	Motoya et al, 2018^14^ (assumed no events and outcomes at week 60 = week 52)
Probability of serious infection over 1 year	0	Motoya et al, 2018^14^ (assumed no infections and outcomes at week 60 = week 52)
Mortality and complications		
Probability of complicated surgical course	0.340	Fazio et al, 2013^16^
Probability of peri-operative mortality	0.001	Fazio et al, 2013^16^
Probability of malignancy-related death	0.035	Hibi et al, 2017^23^ (for GOL)
Probability of death from serious infection	0.001	Lichtenstein et al, 2012^25^
Probability of age-specific, all-cause mortality	0.001	Japanese Ministry of Health, Labor, and Welfare, 2017^27^
Non-Hodgkin lymphoma		
Age- and sex-specific incidence	0.000084	Scott et al, 2018^15^
Baseline hazard with azathioprine use	5.280	Beaugerie et al, 2009^26^
Baseline hazard with TNFα antagonist use	1.000	Beaugerie et al, 2009^26^
Utility estimates		
Baseline	0.320	Arseneau et al, 2006^17^ (assumed same as UC flare)
Remission	0.790	Arseneau et al, 2006^17^
UC in clinical response	0.680	Scott et al, 2018^15^
UC flare	0.320	Arseneau et al, 2006^17^
Serious infection	0.620	Scott et al, 2018^15^
SAE	0.470	Arseneau et al, 2006^17^
Non-Hodgkin lymphoma per cycle	0.118	Lewis et al, 2000^28^; Scott et al, 2015^29^
Immediate postoperative course	0.250	Lewis et al, 2000^28^; Scott et al, 2015^29^
Postoperative remission/pouch	0.680	Arseneau et al, 2006^17^
Disutility estimates		
Complicated surgical course	0.100	Scott et al, 2018^15^

ADA, adalimumab; GOL, golimumab; IBD, inflammatory bowel disease; INF, infliximab; SAE, serious adverse event; TNFα, tumor necrosis factor alpha; UC, ulcerative colitis.

Randomized controlled trials were used to inform transition probabilities, if available. In cases where randomized trials were not available, estimates were obtained from observational studies, systematic reviews/meta-analyses, or expert opinion (in the absence of any published data). Transition probabilities for azathioprine were estimated from 3 non-Japanese (US) publications, including the SUCCESS trial.^15, 18, 19^ The proportion of patients moving directly from azathioprine to the first TNFα antagonist (79.6%) was informed by analysis of the aforementioned cohort of 642 steroid-dependent UC patients from the MDV database. For tacrolimus and cytapheresis, transition probabilities were derived from 2 Japanese observational studies; ^20, 21^ all values except for the probability of flare while in remission over 1 year were assumed to be related to tacrolimus only. The probability of flare while in remission was based on a weighted proportion of the rates for tacrolimus and cytapheresis.^20, 21^ Transition probabilities for TNFα antagonists were estimated using data from 3 Japanese, randomized, double-blind, placebo-controlled trials of adalimumab, infliximab, and golimumab, respectively.^22–24^ An exception was the probability of maintaining remission up to 30 weeks during TNFα antagonist maintenance treatment, which was derived from the prior US Markov model.^15^ Proportional use of tacrolimus vs cytapheresis (66.5% vs 33.5%, respectively; reweighted values excluding other treatments) and adalimumab vs infliximab vs golimumab (43.0% vs 42.6% vs 14.4%, respectively; reweighted values) was informed using the steroid-dependent UC cohort from the MDV database. The recently published, phase 3, randomized, double-blind trial of vedolizumab vs placebo in Japanese UC patients was used to estimate transition probabilities for vedolizumab.^14^ Data from a post-hoc subgroup analysis of the phase 3 trial (reported within the primary publication) allowed stratification of patients according to prior TNFα antagonist use. Algorithms 1 and 2 utilized data for TNFα antagonist-naïve patients, whereas Algorithms 3 and 4 utilized data for TNFα antagonist-exposed patients.

Base-case rates for surgical complications and peri-operative mortaility, mortality due to malignancy (lymphoma) or serious infection, and hazards related to malignancy (lymphoma associated with azathioprine or TNFα antagonist use) were obtained from a combination of US and Japanese publications (Table 1).^16, 23, 25, 26^ All of these risks were modeled independently of each other. The age-specific, all-cause mortality rate (in the base-case scenario, for a 40-year-old Japanese male) was taken from the 2017 Japanese Ministry of Health, Labor, and Welfare life tables.^27^ As Japanese-specific quality-of-life data were not available, all utility values were informed from US publications (Table 1).^15, 17, 28, 29^

Modeled outcomes were quality-adjusted life-years (QALYs; for each treatment pathway), the number of patients in clinical steroid-free remission, the number of cases of malignancy, and the number of cases of serious infection, all expressed per 100,000 patients over the time horizon. QALYs were calculated by multiplying the utlility value for a particular health state by the number of years lived in that state. A QALY value of 1 represents 1 year of life in perfect health; a value of 0 represents death.

### Statistical Methods

The analyses were undertaken using Microsoft Excel 2016 (Redmond, WA, USA). Outcomes were estimated for all 4 algorithms using the base-case scenario over the 1-year time horizon. Incremental QALY benefit was determined as the difference in mean QALY estimates between 2 algorithms. The analyses were repeated under different scenarios where the time horizon was extended (to 3, 5, and 7 years) and alternative discount rates were applied (0%, 4%, and 5%, as used in the US simulation model,^15^ run over a 7-year time horizon).

### Sensitivity Analyses

To test for uncertainty in the model, one-way and probabilistic sensitivity analyses were conducted on the QALY estimates. For the one-way analysis, transition probabilities were varied by 25% and utlities by 15%, with the analysis performed for 1000 patients over the base-case time horizon of 1 year. In this analysis, Algorithm 1 was compared separately with Algorithms 2, 3, and 4, and the data are illustrated as Tornado diagrams. The probabilistic sensitivity analysis was conducted using second-order Monte Carlo simulation in which multiple parameters (based on their distributions) were varied simultaneously for 1000 patients over 500 iterations and a 1-year time horizon. Normal distributions were used for the QALY estimates, with the mean value being the point estimate in the model. A beta distribution was used for the transition probabilities and utility inputs, and a gamma distribution was used for the hazard ratios related to malignancy.

## RESULTS

Under the base-case scenario, Algorithm 1 (vedolizumab administration after azathioprine) was associated with the highest number of expected QALYs (0.453 per 100,000 patients) at the end of the 1-year time horizon when compared with the other algorithms (Table 2). The incremental expected QALY benefit for Algorithm 1 over the other pathways ranged from 0.028 to 0.031 QALYs per 100,000 patients. The predicted number of patients in clinical steroid-free remission over 1 year was similar with Algorithm 1 (32,010 per 100,000 patients) and Algorithm 2 (32,521 per 100,000 patients; vedolizumab administration after azathioprine and tacrolimus/cytapheresis); for both algorithms, the predicted number of patients in remission was higher than that predicted for Algorithm 3 (29,498 per 100,000 patients) and Algorithm 4 (29,445 per 100,000 patients). Across the 4 algorithms over the 1-year horizon, the number of predicted cases of malignancy and serious infection was 17–18 and 119–120 per 100,000 patients, respectively.

**Table 2.** Base-Case Simulations Over a 1-Year Time Horizon

Outcome per 100,000 Patients	Algorithm 1	Algorithm 2	Algorithm 3	Algorithm 4
Expected QALYs	0.453	0.425	0.423	0.422
Individuals in remission	32,010	32,521	29,498	29,445
Cases of malignancy	17	18	18	18
Cases of serious infection	119	119	120	120

QALY, quality-adjusted life year.

**Figure 2.**
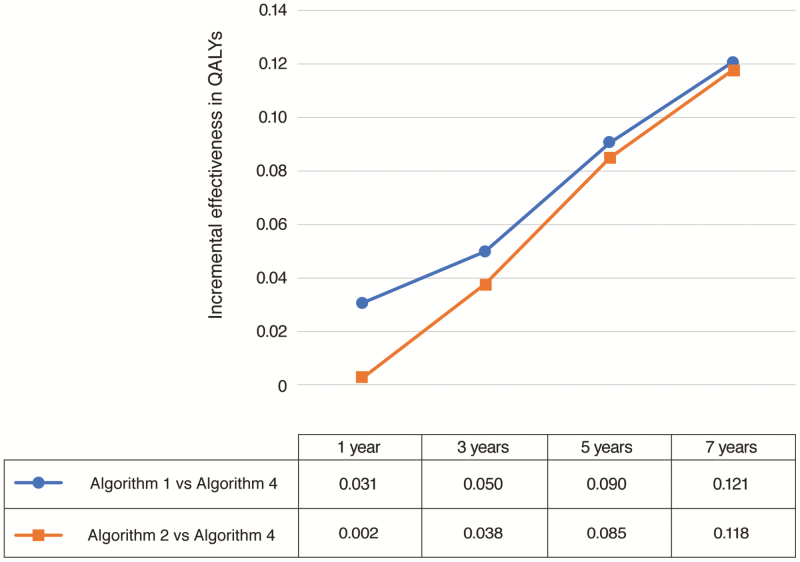
Incremental expected QALY benefit associated with vedolizumab administration as first (Algorithm 1) or second (Algorithm 2) treatment after azathioprine compared with last-line drug therapy (Algorithm 4; immediately before colectomy) from 1 to 7 years. QALY, quality-adjusted life year.

Over extended time horizons, Algorithm 1 remained the preferred strategy in terms of expected QALYs at 3, 5, and 7 years (Table 3). However, at 5 and 7 years, Algorithm 1 and Algorithm 2 were almost equally dominant. As shown in Fig. 2, the incremental expected QALY benefit associated with early (Algorithms 1 and 2) vs late (Algorithm 4) vedolizumab administration increased over longer time horizons, with the greatest benefit seen at 7 years. At 7 years, the maximum incremental expected QALY benefits for Algorithm 1 vs Algorithm 4 and Algorithm 2 vs Algorithm 4 were 0.121 and 0.118 QALYs per 100,000 patients, respectively.

**Table 3.** Expected QALYs Over Base-Case and Extended Time Horizons

	Expected QALYs per 100,000 Patients (95% CI)			
Time Horizon, Years	Algorithm 1	Algorithm 2	Algorithm 3	Algorithm 4
1 (base case)	0.453 (0.453–0.456)	0.425 (0.424–0.427)	0.423 (0.422–0.426)	0.422 (0.422–0.425)
3	1.657 (1.655–1.659)	1.645 (1.643–1.647)	1.588 (1.586–1.590)	1.607 (1.605–1.609)
5	2.705 (2.701–2.709)	2.699 (2.695–2.703)	2.602 (2.598–2.606)	2.614 (2.610–2.619)
7	3.606 (3.600–3.612)	3.603 (3.597–3.609)	3.484 (3.477–3.490)	3.485 (3.479–3.492)

CI, confidence interval; QALY, quality-adjusted life year.

The effect of varying the discount rate on incremental QALY benefit over a 7-year time horizon is shown in Supplementary Table S1. As the discount rate increased, the expected total QALYs per 100,000 patients for Algorithm 1 decreased. In terms of incremental QALY benefit per 100,000 patients, Algorithm 1 was the preferred strategy when discount rates of 0%, 4%, and 5% were applied (0.011–0.013 vs Algorithm 2, 0.100–0.111 vs Algorithm 3, and 0.096–0.108 vs Algorithm 4).

### Sensitivity Analyses

**Figure 3.**
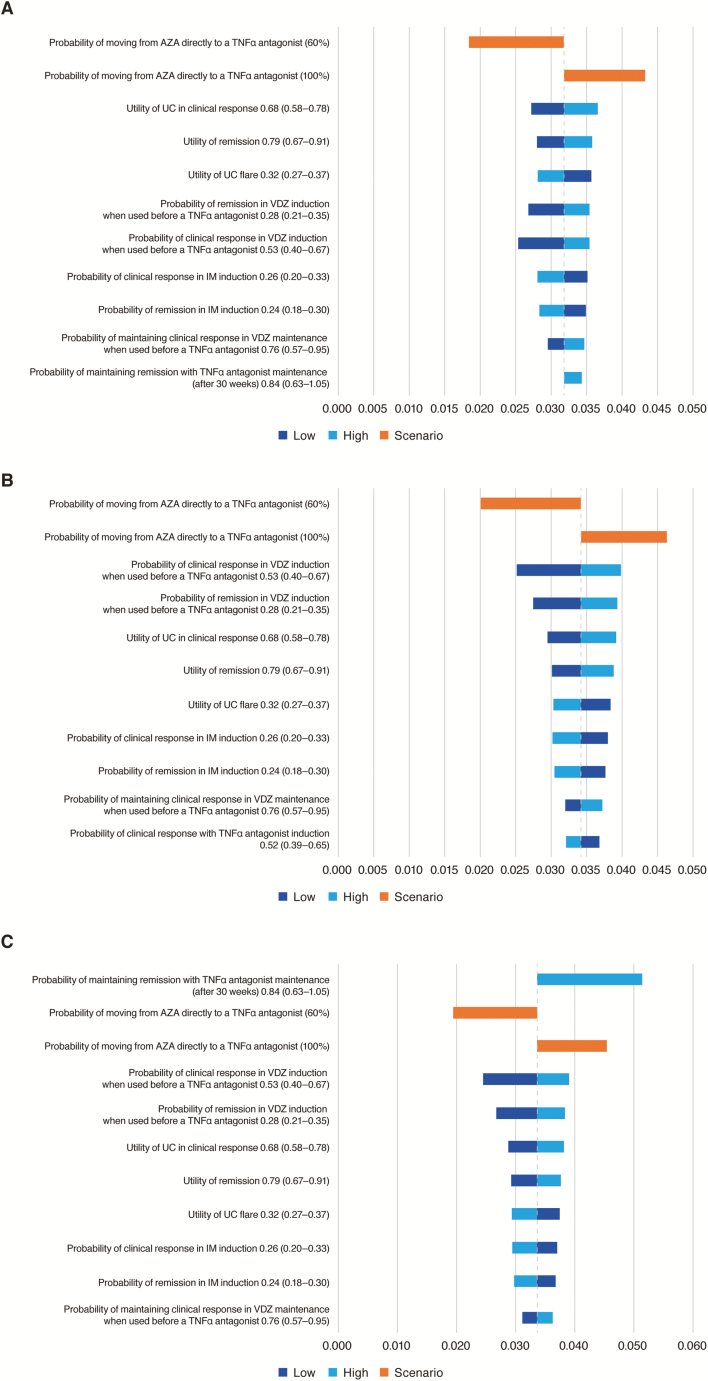
One-way sensitivity analysis (Tornado diagrams) showing the effect of varying model input parameters on the incremental expected QALY benefit associated with vedolizumab administration as first treatment after azathioprine (Algorithm 1) compared with vedolizumab administration as: (A) second treatment after azathioprine (Algorithm 2); (B) third treatment after azathioprine (Algorithm 3); and (C) last-line drug therapy (Algorithm 4, immediately before colectomy) over a 1-year time horizon. AZA, azathioprine; IM, immunomodulator; QALY, quality-adjusted life year; TNFα, tumor necrosis factor alpha; UC, ulcerative colitis.

In the one-way sensitivity analysis comparing Algorithm 1 with Algorithm 2, the probability of moving azathioprine-treated patients directly to the first TNFα antagonist was the most impactful parameter on expected incremental expected QALY benefit (Fig. 3A). Utility values associated with clinical response, remission, and UC flare were the next most impactful parameters. For the comparison of Algorithm 1 with Algorithm 3, the parameters that impacted most on incremental expected QALY benefit were the probability of moving azathioprine-treated patients directly to the first TNFα antagonist, the probability of clinical response or remission during vedolizumab induction when administered before a TNFα antagonist, and utility values associated with clinical response and remission (Fig. 3B). When comparing Algorithm 1 with Algorithm 4, the most impactful parameters were the probability of maintaining remission during TNFα antagonist maintenance therapy, the probability of moving azathioprine-treated patients directly to the first TNFα antagonist, and the probability of clinical response or remission during vedolizumab induction when administered before a TNFα antagonist (Fig. 3B).

The probabilistic sensitivity analyses of the expected QALY values showed close alignment with the base-case results, with Algorithm 1 remaining dominant over the other 3 treatment strategies 100% of the time (Table 4).

**Table 4.** Probabilistic Sensivity Analysis Over A 1-Year Time Horizon

Expected QALYs per 100,000 Patients	Algorithm 1	Algorithm 2	Algorithm 3	Algorithm 4
Mean value	0.452	0.425	0.423	0.422
Mean 95% CI	(0.453–0.456)	(0.424–0.427)	(0.422–0.426)	(0.422–0.425)
Mean incremental benefit	–	0.029	0.030	0.031
Incremental benefit 95% CI	–	(0.028–0.029)	(0.030–0.031)	(0.031–0.031)

CI, confidence interval; QALY, quality-adjusted life year.

## DISCUSSION

Vedolizumab was approved by the Japanese Ministry of Health, Labor, and Welfare in July 2018 for the treatment of adult patients with moderate-to-severely active UC who have had an inadequate response with, lost response to, or were intolerant to a TNFα antagonist or immunomodulator, or had an inadequate response with, were intolerant to, or demonstrated dependence on corticosteroids.^12^ While this indication encompasses steroid-dependent UC, there is no defined algorithm for use of biologic agents in Japanese treatment guidelines and it remains unclear where new therapies, such as vedolizumab, fit in to current recommendations for these patients.^1^ Therefore, in the absence of clinical trials comparing treatment sequences, we constructed a Japanese-specific Markov simulation model to predict where, in the current Japanese treatment paradigm, vedolizumab might provide the optimal clinical benefit (in terms of QALYs) for patients with moderate-to-severely active, steroid-dependent UC in need of steroid-sparing therapy.

The current model, which incorporated Japanese data wherever possible and reflects current treatment practice in Japan,^1^ predicted that an algorithm where vedolizumab is introduced as second-line therapy (ie, after azathioprine; Algorithm 1) may provide the optimal clinical benefit for patients, in terms of QALYs, over the course of 1 year. Furthermore, clinical trial data suggest that response and remission rates to a second TNFα antagonist may be reduced after an initial failure.^30^ However, a systematic review of Japanese literature, conducted as part this study, found no evidence to support this. Therefore, the efficacy of first or second TNFα antagonist was assumed to be equivalent in the present analysis.

Tacrolimus/cytapheresis are therapies commonly used for the management of UC in Japan; yet little data are available for inclusion in this model. For example, the probability of SAE applied to this model was 0% in our literature search, but this will need to be updated as more data from clinical studies for tacrolimus/cytapheresis become available. Besides, scenario analyses over extended time horizons predicted that this strategy would remain dominant over a 7-year period. It should be highlighted that, at 5 and 7 years, a similar QALY benefit was predicted for the algorithm where vedolizumab is introduced as third-line therapy (ie, after azathioprine and tacrolimus/cytapheresis but before other biologic therapy; Algorithm 2). The greater predicted QALY benefit observed with early introduction of vedolizumab (as second- or third-line treatment) compared with later administration can be attributed to the higher clinical remission rates that are predicted when the drug is administered before a TNFα antagonist (as shown in Table 2).

Overall, these findings suggest that vedolizumab should not be restricted to use after failure or intolerance of TNFα antagonists or tacrolimus/cytapheresis in Japanese patients with moderate-to-severely active, steroid-dependent UC, requiring steroid-sparing therapy. The results also indicate that the optimal clinical benefit is observed when vedolizumab is introduced as first treatment after loss of response or remission to an immunomodulator (in this case, azathioprine), thus indicating the value of early intervention. Previous post hoc analyses of the GEMINI 1 study and the Japanese phase 3 trial have also suggested that early use of vedolizumab may be clinically beneficial.^13, 14^ In both randomized, placebo-controlled studies, numerically higher rates of clinical response (and remission in GEMINI 1) to vedolizumab treatment were observed in TNFα antagonist-naive vs -exposed patients. Consistently, observational studies have also demonstrated the efficacy of vedolizumab in patients who have not been exposed to prior TNFα antagonists.^31–33^

As stated earlier, the model developed for this Japanese-specific analysis was based on a US Markov simulation model that evaluated the optimal position of vedolizumab in the UC treatment pathway from a US perspective.^15^ While the 2 models share a comparable basic structure and demonstrated similar results, our construct included the use of tacrolimus/cytapheresis, reflecting Japanese clinical practice.^1^ The present model also incorporated Japanese data for the clinical inputs (if available), which may explain the slightly smaller expected QALY benefits compared with the US model.^15^

Regardless of which algorithm was simulated in the 1-year base-case scenario, there were few predicted cases of malignancy (17–18 per 100,000 patients) and the number of predicted cases of serious infection was low (119–120 per 100,000 patients). These findings are consistent with the published literature on the safety of vedolizumab (which describes low incidence rates of serious infections and malignancies over an extended treatment period),^34^ and suggest that adverse outcomes linked with long-term immunosuppressive or biologic therapy^35^ are likely to be unaffected by the positioning of vedolizumab in the Japanese UC treatment pathway.

The model used in the present study was based on the latest and best available evidence, and the sensitivity analyses demonstrated the robustness of the data. Advanced mathematical modeling techniques were used to determine the optimal position for vedolizumab introduction in the Japanese treatment paradigm. The model mimicked variations in clinical practice by altering the order of treatment options (which included the most widely used treatments in Japan) and utilizing clinical variables that account for prior treatment exposure. It also accounted for the different responses to vedolizumab in patients with prior exposure to TNFα antagonists.^14^ However, due to a lack of data, multiple assumptions had to be made (as indicated in Table 1) to account for differences in study design and duration, patient populations, and missing data. In addition, because of the absence of suitable quality-of-life studies in Japan, Japanese-specific utility data were not available, and consequently US data had to be inputted. However, there may be ethnic and cultural differences in the preferences for specific health-related outcomes between Japanese and US patients. Another limitation relates to the lack of published data describing the impact of prior vedolizumab administration on the subsequent effectiveness of TNFα antagonists. Notably, the sensitivity analyses showed that early vedolizumab administration was still the preferred strategy over a 1-year horizon after a ≤25% absolute reduction in TNFα antagonist efficacy. Although not modeled in our analysis, over longer time horizons, the effect of prior vedolizumab administration on TNFα antagonist efficacy may have a greater impact on the preferred treatment strategy. A further limitation of the analysis relates to the estimation of transition probabilities from the MDV database. As the MDV database contains mainly Diagnosis Procedure Combination data collected from acute hospitals, the findings cannot necessarily be applied to all other clinical settings in Japan. Lastly, because of limited data, our model did not account for mucosal healing, which is an increasingly important endpoint in clinical trials, but is not always monitored in clinical practice. Evaluation of the relative impact of the different treatment approaches in steroid-refractory patients or on healthcare costs was beyond the scope of this analysis but could be the subject of future analyses.

In conclusion, this Markov simulation analysis predicts that administering vedolizumab as first treatment after a thiopurine may provide the optimal clinical benefit for Japanese patients with steroid-dependent moderate-to-severely active UC, in terms of QALYs. There were no differences among any of the 4 algorithms for the probability of serious infection or malignancy (lymphoma). Prospective studies are required to directly compare sequential treatment pathways for UC. If cost inputs were to be considered, our model framework could also be used in the future to evaluate the cost-effectiveness of alternative treatment strategies in steroid-dependent UC.

## Supplementary Material

otaa017_suppl_Supplementary_Material

## Notes

Funding: This research was funded by Takeda Pharmaceutical Co. Ltd.

Conflict of interest disclosure: Y.L., S.D., and H.M .have acted as consultants for Takeda Pharmaceutical Co. Ltd. through their employment at IQVIA Solutions. A.U., Y.E., T.W., M.O., and R.I. are employees of Takeda Pharmaceutical Co. Ltd. A.I. has received consulting fees from Novartis Pharma KK, AbbVie GK, Milliman, Sony Inc., and Eli Lilly Japan KK; lecture fees from Chugai Pharmaceutical Company, Ltd., CRECON Research and Consulting Inc., Terumo Corporation, Bristol-Myers Squibb KK, and Creativ Ceutical KK; transport fees from Takeda Pharmaceutical Co. Ltd.; and research grants from Pfizer Japan Inc., CSL Behring Japan Inc., Gilead Science KK, and Fuji Film KK. All authors had a role in designing the study, analyzing and interpreting the data, and preparing the manuscript and critically revising it for important intellectual content. The final draft of the manuscript was approved by all authors before submission.

## References

[1] Matsuoka K , Kobayashi T, Ueno F, et al. Evidence-based clinical practice guidelines for inflammatory bowel disease. J Gastroenterol. 2018;53:305–353. 29429045 10.1007/s00535-018-1439-1 PMC5847182

[2] Ordás I , Eckmann L, Talamini M, et al. Ulcerative colitis. Lancet. 2012;380:1606–1619. 22914296 10.1016/S0140-6736(12)60150-0

[3] Ng SC , Shi HY, Hamidi N, et al. Worldwide incidence and prevalence of inflammatory bowel disease in the 21st century: a systematic review of population-based studies. Lancet. 2018;390:2769–2778. 10.1016/S0140-6736(17)32448-0 29050646

[4] Ng WK , Wong SH, Ng SC. Changing epidemiological trends of inflammatory bowel disease in Asia. Intest Res. 2016;14:111–119. 27175111 10.5217/ir.2016.14.2.111 PMC4863044

[5] Japanese Ministry of Health Labor and Welfare. The 26th year of my life. http://www.mhlw.go.jp/toukei/saikin/hw/eisei_houkoku/14/dl/kekka7.pdf (10 June 2019, date last accessed).

[6] Wehkamp J , Stange EF. Recent advances and emerging therapies in the non-surgical management of ulcerative colitis. F1000Res. 2018;7:1207. 10.12688/f1000research.15159.1 PMC6081982 30135722

[7] Roda G , Jharap B, Neeraj N, Colombel JF. Loss of response to Anti-TNFs: definition, epidemiology, and management. Clin Transl Gastroenterol. 2016;7:e135. 26741065 10.1038/ctg.2015.63 PMC4737871

[8] Soler D , Chapman T, Yang LL, et al. The binding specificity and selective antagonism of vedolizumab, an anti-alpha4beta7 integrin therapeutic antibody in development for inflammatory bowel diseases. J Pharmacol Exp Ther. 2009;330:864–875. 19509315 10.1124/jpet.109.153973

[9] Feagan BG , Rutgeerts P, Sands BE, et al.; GEMINI 1 Study Group. Vedolizumab as induction and maintenance therapy for ulcerative colitis. N Engl J Med. 2013;369:699–710. 23964932 10.1056/NEJMoa1215734

[10] European Medicines Agency. ENTYVIO (vedolizumab) EU summary of product characteristics. Takeda Pharma A/S. Last updated: 4 January 2019. https://www.ema.europa.eu/en/documents/product-information/entyvio-epar-product-information_en.pdf (10 June 2019, date last accessed).

[11] U.S. Food and Drug Administration. ENTYVIO (vedolizumab) US prescribing information. Takeda Pharmaceuticals America Inc. Revised: 05/2019. https://general.takedapharm.com/ENTYVIOPI (10 June 2019, date last accessed).

[12] Pharmaceuticals and Medical Devices Agency. ENTYVIO (vedolizumab) product insert [in Japanese]. Takeda Pharmaceutical Company Ltd. Last updated: May 22, 2019. http://www.pmda.go.jp/PmdaSearch/iyakuDetail/ResultDataSetPDF/400256_2399405F1020_1_03 (10 June 2019, date last accessed).

[13] Feagan BG , Rubin DT, Danese S, et al. Efficacy of Vedolizumab Induction and Maintenance Therapy in Patients With Ulcerative Colitis, Regardless of Prior Exposure to Tumor Necrosis Factor Antagonists. Clin Gastroenterol Hepatol. 2017;15:229–239.e5. 27639327 10.1016/j.cgh.2016.08.044

[14] Motoya S , Watanabe K, Ogata H, et al. Vedolizumab in Japanese patients with ulcerative colitis: a Phase 3, randomized, double-blind, placebo-controlled study. PLoS One. 2019;14:e0212989. 30807613 10.1371/journal.pone.0212989 PMC6391030

[15] Scott FI , Shah Y, Lasch K, et al. Assessing the optimal position for vedolizumab in the treatment of ulcerative colitis: a simulation model. Inflamm Bowel Dis. 2018;24:286–295. 29361100 10.1093/ibd/izx045

[16] Fazio VW , Kiran RP, Remzi FH, et al. Ileal pouch anal anastomosis: analysis of outcome and quality of life in 3707 patients. Ann Surg. 2013;257:679–685. 23299522 10.1097/SLA.0b013e31827d99a2

[17] Arseneau KO , Sultan S, Provenzale DT, et al. Do patient preferences influence decisions on treatment for patients with steroid-refractory ulcerative colitis? Clin Gastroenterol Hepatol. 2006;4:1135–1142. 16829206 10.1016/j.cgh.2006.05.003

[18] Panaccione R , Ghosh S, Middleton S, et al. Combination therapy with infliximab and azathioprine is superior to monotherapy with either agent in ulcerative colitis. Gastroenterology. 2014;146:392–400.e3. 24512909 10.1053/j.gastro.2013.10.052

[19] Timmer A , McDonald JW, Tsoulis DJ, et al Azathioprine and 6-mercaptopurine for maintenance of remission in ulcerative colitis. Cochrane Database Syst Rev. 2012:CD000478. 22972046 10.1002/14651858.CD000478.pub3

[20] Yamamoto T , Shimoyama T, Umegae S, Matsumoto K. Tacrolimus vs. anti-tumour necrosis factor agents for moderately to severely active ulcerative colitis: a retrospective observational study. Aliment Pharmacol Ther. 2016;43:705–716. 26762838 10.1111/apt.13531

[21] Yokoyama Y , Matsuoka K, Kobayashi T, et al. A large-scale, prospective, observational study of leukocytapheresis for ulcerative colitis: treatment outcomes of 847 patients in clinical practice. J Crohns Colitis. 2014;8:981–991. 24556083 10.1016/j.crohns.2014.01.027

[22] Suzuki Y , Motoya S, Hanai H, et al. Efficacy and safety of adalimumab in Japanese patients with moderately to severely active ulcerative colitis. J Gastroenterol. 2014;49:283–294. 24363029 10.1007/s00535-013-0922-y PMC3925299

[23] Hibi T , Imai Y, Senoo A, et al. Efficacy and safety of golimumab 52-week maintenance therapy in Japanese patients with moderate to severely active ulcerative colitis: a phase 3, double-blind, randomized, placebo-controlled study-(PURSUIT-J study). J Gastroenterol. 2017;52:1101–1111. 28324167 10.1007/s00535-017-1326-1 PMC5606947

[24] Suzuki Y , Motoya S, Hirai F, et al P584 Infliximab therapy for Japanese patients with ulcerative colitis: efficacy, safety, and association between serum infliximab levels and early response in a randomized, double-blind, placebo-controlled study. Journal of Crohn’s and Colitis. 2015;9:S372–S373.

[25] Lichtenstein GR , Rutgeerts P, Sandborn WJ, et al. A pooled analysis of infections, malignancy, and mortality in infliximab- and immunomodulator-treated adult patients with inflammatory bowel disease. Am J Gastroenterol. 2012;107:1051–1063. 22613901 10.1038/ajg.2012.89 PMC3390465

[26] Beaugerie L , Brousse N, Bouvier AM, et al.; CESAME Study Group. Lymphoproliferative disorders in patients receiving thiopurines for inflammatory bowel disease: a prospective observational cohort study. Lancet. 2009;374:1617–1625. 19837455 10.1016/S0140-6736(09)61302-7

[27] Japanese Ministry of Health Labor and Welfare. Life tables 2017. https://www.mhlw.go.jp/english/database/db-hw/vs02.html (10 June 2019, date last accessed).

[28] Lewis JD , Schwartz JS, Lichtenstein GR. Azathioprine for maintenance of remission in Crohn’s disease: benefits outweigh the risk of lymphoma. Gastroenterology. 2000;118:1018–1024. 10833475 10.1016/s0016-5085(00)70353-2

[29] Scott FI , Vajravelu RK, Bewtra M, et al The benefit-to-risk balance of combining infliximab with azathioprine varies with age: a markov model. Clin Gastroenterol Hepatol. 2015;13:302–309 e311. 25117775 10.1016/j.cgh.2014.07.058 PMC4324381

[30] Gisbert JP , Marín AC, McNicholl AG, Chaparro M. Systematic review with meta-analysis: the efficacy of a second anti-TNF in patients with inflammatory bowel disease whose previous anti-TNF treatment has failed. Aliment Pharmacol Ther. 2015;41:613–623. 25652884 10.1111/apt.13083

[31] Kopylov U , Verstockt B, Biedermann L, et al. Effectiveness and Safety of Vedolizumab in Anti-TNF-Naïve Patients With Inflammatory Bowel Disease-A Multicenter Retrospective European Study. Inflamm Bowel Dis. 2018;24:2442–2451. 29788318 10.1093/ibd/izy155

[32] Allamneni C , Venkata K, Yun H, et al. Comparative effectiveness of vedolizumab vs. Infliximab induction therapy in ulcerative colitis: experience of a real-world cohort at a tertiary inflammatory bowel disease center. Gastroenterology Res. 2018;11:41–45. 29511405 10.14740/gr934w PMC5827901

[33] Loftus EV Jr , Colombel JF, Feagan BG, et al. Long-term efficacy of vedolizumab for ulcerative colitis. J Crohns Colitis. 2017;11:400–411. 27683800 10.1093/ecco-jcc/jjw177

[34] Colombel JF , Sands BE, Rutgeerts P, et al. The safety of vedolizumab for ulcerative colitis and Crohn’s disease. Gut. 2017;66:839–851. 26893500 10.1136/gutjnl-2015-311079 PMC5531223

[35] Holmer A , Singh S. Overall and comparative safety of biologic and immunosuppressive therapy in inflammatory bowel diseases. Expert Rev Clin Immunol. 2019:1–11. 10.1080/1744666X.2019.1646127 PMC6813772 31322018

