# Evaluation of the Optimal Position for Vedolizumab in the Japanese Treatment Paradigm for Ulcerative Colitis Using Markov Modeling

**DOI:** 10.1093/crocol/otaa036

**Published:** 2020-05-30

**Authors:** Akihito Uda, Yuki Eto, Yuxin Li, Hiroyuki Matsuda, Sven Demiya, Tomoyuki Watanabe, Mihoko Ota, Ryuichi Iwakiri, Ataru Igarashi

In Figure 1 of the initially published article, the direction of four arrows and the shape of three objects in the Markov simulation model, indicated in the attached file with red circles, were incorrectly shown. Figure 1 has been corrected to show the accurate model used in the study. These corrections have not changed the findings of the article.

