# Supplementary material for: Evaluation of the Optimal Position for Vedolizumab in the Japanese Treatment Paradigm for Ulcerative Colitis Using Markov Modeling
Source: Crohns Colitis 360. 2020 Mar 18;2(2):otaa017. doi: 10.1093/crocol/otaa017 (PMC9802218; doi:10.1093/crocol/otaa017)
Supplement: otaa017_suppl_Supplementary_Material [file otaa017_suppl_supplementary_material.docx]

**Supplementary TABLES**

SUPPLEMENTARY TABLE S1. Expected QALYs for vedolizumab administration as first treatment after azathioprine (Algorithm 1) and incremental expected QALY benefit compared with later use (Algorithms 2–4) according to discount rate over a 7-year time horizon.

|  | Expected QALYs per 100,000 patients |  | Incremental QALY benefit per  100,000 patients | | |
| --- | --- | --- | --- | --- | --- |
| Discount rate | Algorithm 1 |  | Algorithm 2 | Algorithm 3 | Algorithm 4 |
| 0% | 3.823 |  | 0.011 | 0.111 | 0.108 |
| 4% | 3.415 |  | 0.012 | 0.102 | 0.098 |
| 5% | 3.325 |  | 0.013 | 0.100 | 0.096 |

QALY, quality-adjusted life year.

# Supplementary Digital Content 1: JAPANESE systematic literature review

## Objectives

A systematic literature review was undertaken to identify Japanese-specific clinical variables (transition probabilities) for vedolizumab and its comparators, along with outcome estimates to inform the Japanese-specific Markov simulation model.

The objectives of the systematic review were as follows:

1. For vedolizumab and its comparators, to find the probability of…

- remission during induction
- a clinical response during induction
- a serious adverse event (SAE) during induction
- a serious infection during induction
- maintaining remission
- maintaining a clinical response
- a flare with clinical response
- an SAE requiring discontinuation of therapy with maintenance therapy
- a serious infection while on maintenance therapy
- mucosal healing.

2. To estimate mortality rates for…

- malignancy-related deaths
- death from serious infection
- age-specific, all-cause mortality for the average Japanese patient with ulcerative colitis (UC).

3. To identify UC-specific quality of life (QoL) utility estimates for…

- remission
- UC in clinical response
- a UC flare
- a serious infection
- an SAE
- malignancy per cycle
- immediate post-operative course
- postoperative remission/pouch
- disutility for a complicated surgical course.

## Methods

The systematic review was conducted using both English and Japanese databases, supplemented with hand searches of the ‘gray’ literature. Published reports of randomized controlled trials, observational studies, systematic reviews, or meta-analyses were identified by searching the MEDLINE (PubMed), EMBASE, and Ichu-shi (Japanese language) literature databases. The searches were restricted to English- or Japanese-language publications originating from Japan, published between January 1, 2000 and January 29, 2019 (the date of the search). The most recent mortality data were identified using the Japanese Ministry of Health, Labor, and Welfare (<https://www.mhlw.go.jp/toukei_hakusho/index.html>) and e-Stat (portal site of the Official Statistics of Japan; <https://www.e-stat.go.jp/>) websites. Utility (QoL) data were searched for in the literature databases, National Institute for Health and Care Excellence (NICE) technology appraisals (<https://www.nice.org.uk/>), the Niigata University of Health and Welfare Center for Health Economics & QOL Research website (<http://cheqol.com/index.html>), and the Tufts University Center for the Evaluation of Value and Risk in Health Cost-Effectiveness Analysis (CEA) registry (<https://cevr.tuftsmedicalcenter.org/databases/cea-registry>).

The scope of the review was defined in terms of patients, interventions, comparators, outcomes, and study type (ie, PICOS statement; Table A1).

TABLE A1. PICOS statement.

| Criteria | Definition |
| --- | --- |
| Patient population | Adult patients (aged ≥18 years) with moderate-to-severely active UC in Japan |
| Intervention | Prednisolone, corticosteroids, glucocorticoids, steroids, azathioprine, 6-MP, 5-ASA, mesalazine, tacrolimus, ciclosporin, TNFα antagonist, infliximab, adalimumab, golimumab, anti-α_4_β_7_ integrin, vedolizumab, cytapheresis, colectomy, surgery (monotherapy or combination therapy [≥2 drugs]) |
| Comparators | Any treatment from the above-mentioned list of interventions (monotherapy or combination therapy [≥2 drugs]) |
| Outcome measures | Clinical  Remission, clinical response, AE(s), SAE(s), infection, flare, discontinuation, surgical complications  Safety  Mortality, surgical mortality, malignancy-related death, infection-related death, death  *QoL*   - QoL (disease or non-disease specific, eg, IBDQ, SF-36, etc.), HRQoL, functional status, well-being scores AND/OR - Utility values elicited using the following techniques: TTO, SG, generic preference-based instruments (eg, EQ-5D, SF-36) AND/OR - Utilities derived from disease specific measures (eg, IBDQ) |
| Study design | RCTs OR observational studies OR systematic review OR meta-analyses |
| Restrictions | English/Japanese language  Year limit: 2000–2019  Countries: Japan |

5-ASA, 5-aminosalicylic acid; 6-MP, 6-mercaptopurine; AE, adverse event; EQ-5D, EuroQol-5D; HRQoL, health-related quality of life; IBDQ, Inflammatory Bowel Disease Questionnaire; QoL, quality of life; RCTs, randomized controlled trials; SAE, serious adverse event; SF-36, 36-Item Short Form Survey; SG, standard gamble; TNFα, tumor necrosis factor alpha; TTO, time trade-off; UC, ulcerative colitis.

Once the criteria had been specified (as in Table A1), they were translated into ‘search strings’, after which the searches were executed. Search strategies were based on the title, abstract, and indexing terms (eg, MeSH), and their relationship (using Boolean terms, such as ‘AND, ‘OR’, and ‘NOT’). Citations identified from each database were managed using EndNote X8 (Clarivate Analytics, Philadelphia, PA, USA) and then exported to a Microsoft Excel 2016 (Redmond, WA, USA) spreadsheet for review, after excluding all obvious duplicates.

Publications identified during the search were screened by one researcher against pre-specified selection criteria (Table A2), first by abstract and title, and then by full text. Uncertainty regarding inclusion of a publication was resolved through ‘reconciliation’ (discussion with another reviewer). As an extra quality-control step, 10% of the publications were screened at random by a second reviewer to check for eligibility against the selection criteria. Details of the screening process and reasons for inclusion or exclusion of a publication were recorded.

TABLE A2. Publication selection criteria.

| PICOS | Inclusion criteria | Exclusion criteria |
| --- | --- | --- |
| Population | Adult patients (≥18 years) with moderate-to-severely active UC in Japan | - Non-human studies - Children/pediatric population - Conditions other than UC - All interventions not listed in the inclusion criteria |
| Intervention/comparator | Prednisolone, corticosteroids, glucocorticoids, steroids, azathioprine, 6-MP, 5-ASA, mesalazine, tacrolimus, TNFα antagonist, infliximab, adalimumab, golimumab, anti- α_4_β_7_ integrin, vedolizumab, cytapheresis, ciclosporin, colectomy, surgery | - All interventions not listed in the inclusion criteria |
| Outcome | Probability of:   - remission during induction - a clinical response during induction - an SAE during induction - a serious infection during induction - maintaining remission - maintaining a clinical response - a flare with clinical response - an SAE requiring discontinuation of maintenance - a serious infection while on maintenance - malignancy-related deaths - death from serious infection - age-specific, all-cause mortality for the average Japanese patient with UC developing malignancy | - Any studies not providing specific detail on the outcomes of interest |
|  | Mortality rates for:   - malignancy-related deaths - death from serious infection - age-specific, all-cause mortality for the average Japanese patient with UC |  |
|  | UC-specific QoL estimates for:   - remission - UC in clinical response - UC flare - serious infection - SAE - malignancy per cycle - immediate postoperative course - postoperative remission/pouch - disutility for complicated surgical course |  |
| Study design | - RCTs - Systematic reviews - Meta-analyses - Observational studies | - Any type of studies not listed in the inclusion criteria |
| Restrictions | - Language: English and Japanese - Year limit: 2000–2019 - Countries: Japan | - Any studies that are not in English or Japanese |

5-ASA, 5-aminosalicylic acid; 6-MP, 6-mercaptopurine; QoL, quality of life; RCTs, randomized controlled trials; SAE, serious adverse event; TNFα, tumor necrosis factor alpha; UC, ulcerative colitis.

Information from included publications and data sources was extracted into a standardized (predesigned, tested, and refined) spreadsheet template in Microsoft Excel. Information on study design, patient demographics and clinical characteristics, treatment patterns, clinical and safety outcomes, and QoL was captured (Table A3). The data extraction was performed in English by one researcher and checked by an independent reviewer. Any disagreements were resolved by another independent reviewer. Prior to extraction, data from the Japanese literature were translated into English by a bilingual reviewer.

TABLE A3. Study and patient characteristics, and outcomes included in the data extraction.

| Topic | Data to be extracted |
| --- | --- |
| Published source | - Author(s) - Title - Journal/proceeding - Year - PubMed ID |
| Study overview | - Study objectives - Study country/countries/region - Study design and methods - Number of patients screened and included - Interventions (including drug, dosage, and administration) - Comparators - Study endpoints - Duration of follow-up |
| Patient demographic characteristics | - Age - Gender - Weight |
| Patient clinical characteristics | - Disease phenotype - Age at diagnosis - Time since symptom initiation - Time since diagnosis (disease duration) - Disease location - Disease behavior - Clinical disease activity - Endoscopic disease activity |
| Treatment patterns | - Non-biologic treatment history (including 5-ASA, corticosteroids, and immunomodulators) - Biologic treatment history - Surgical treatment history - Current non-biologic and/or biologic treatment (including dose strength and frequency of administration, duration of treatment) - Dose escalation, dose de-escalation, discontinuation and/or switching of treatment (and reason for this) |
| Clinical outcomes by treatment and follow-up time (including timings and relevant definitions) | - Clinical response - Corticosteroid-free response - Sustained/durable response - Clinical remission - Corticosteroid-free remission - Sustained/durable remission - Corticosteroid reduction/tapering - Endoscopic improvement - Endoscopic remission/mucosal healing - Deep remission (remission + mucosal healing) - Clinical disease activity (eg, Mayo score) - Relapse outcomes |
| QoL | - QoL scores over time - Change from baseline - Differences in change - Methods for deriving utilities - Utility value by health state - AE-related (dis)utility values - Associated utility statistics |
| Safety outcomes by treatment and follow-up time | - AEs - SAEs - Discontinuation due to AEs - Infections/serious infections - Malignancy |

AE, adverse event; QoL, quality of life; SAE, serious adverse event.

## Results

A total of 160 records were identified from the literature database searches and one record was identified through other sources. After removal of duplicates, 148 unique records were identified (Fig. A1). The abstract review identified 78 potentially relevant records for full-text review, of which 42 fulfilled the selection criteria and were included in the review. One additional record identified by additional handsearching, which was published after the timepoint the review was conducted, was also included.

FIGURE A1. PRISMA flow diagram for clinical and quality of life evidence.
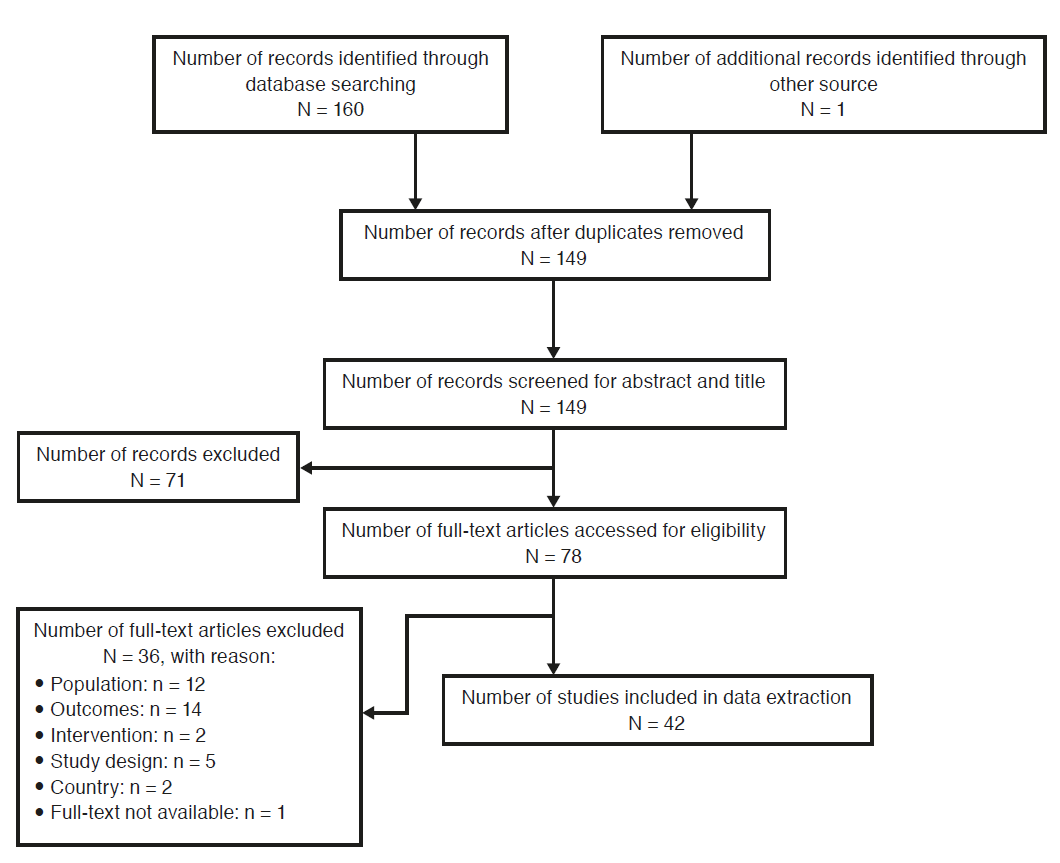


Of the 43 included studies, 40 reported clinical (efficacy and safety) data and three reported QoL data (Inflammatory Bowel Disease Questionnaire [IBDQ] and/or daily life satisfaction score) for Japanese patients with moderately-to-severely active UC. The 40 clinical records included 36 journal articles and four congress abstracts. Of the 40 clinical studies, 10 were randomized controlled trials.

Included studies identified through the MEDLINE/EMBASE, Ichu-shi, and hand searches are shown in Tables A4, A5, and A6, respectively. These studies were used to inform the Japanese-specific Markov model that aimed to define the optimal position of vedolizumab in the treatment paradigm for Japanese patients with moderately-to-severely active UC.

TABLE A4. Overview of included studies (MEDLINE/EMBASE databases).

| Authors, year | Study design  (language) | Intervention | Efficacy | Safety | QoL | Mortality |
| --- | --- | --- | --- | --- | --- | --- |
| Articles | | | | | | |
| Hibi et al., 2017^1^  (PURSUIT-J study) | - Phase 3, RCT, double-blind study (English) | - Golimumab - Placebo | *Induction phase*   - Response - Remission - Mucosal healing   *Maintenance phase*   - Response - Remission - Mucosal healing | - SAE - Infection - SAE requiring discontinuation - Malignancy | NR | Reported |
| Yokoyama et al., 2014^2^ | - PMS (English) | - LCAP | - Remission | NR | NR | NR |
| Nakano et al., 2013^3^ | - Single-center RCT (English) | - GMA - LCAP | - Response - Remission | - SAE | NR | NR |
| Inoue et al., 2013^4^ | - Retrospective, observational study (English) | - Tacrolimus | - Response - Remission - Mucosal healing | - Infection | NR | NR |
| Takayama et al., 2013^5^ | - Retrospective, observational study (English) | - Cytapheresis | - Remission | NR | NR | NR |
| Hibi et al., 2009^6^ | - PMS (English) | - LCAP | - Remission | - SAE | NR | NR |
| Sakata et al., 2008^7^ | - Single-center RCT (English) - Steroid-dependent and steroid-refractory patients included | - LCAP - GMA | - Response - Remission | - SAE | NR | NR |
| Sakuraba et al., 2008^8^ | - Open-label RCT (English) | - GMA | - Remission | - SAE - Infection - SAE requiring discontinuation | NA | NR |
| Matsumoto et al., 2008^9^ | - Multicenter, prospective, open-label study (English) - Steroid-dependent and steroid-refractory patients included | - LCAP | - Response - Remission | NR | NR | NR |
| Ando et al., 2005^10^ | - RCT (English) - All patients are steroid refractory | - LCAP - Ciclosporin | - Response | NR | NR | NR |
| Naganuma et al., 2004^11^ | - Prospective, observational study (English) - Stratified by steroid-dependent and steroid-refractory patients | - GMA | - Response - Remission | - SAE - Malignancy | NR | NR |
| Jo et al., 2003^12^ | - Retrospective, observational study (English) | - Prednisolone + LCAP - Prednisolone | - Response - Remission | - SAE | NR | NR |
| Yokoyama et al., 2015^13^ | - Prospective, multicenter study (English) | - GMA | *Induction phase*   - Response - Remission   *Maintenance phase*   - Remission | NR | - IBDQ |  |
| Conference abstracts | | | | | | |
| Suzuki et al., 2015^14^ | - Double-blind RCT (English) | - Infliximab - Placebo | - Response - Remission - Mucosal healing | - SAE | NR | NR |
| Nuki et al., 2013^15^ | - Retrospective survey (English) | - Infliximab - Tacrolimus | - Response - Remission | NR | NR | NR |
| Fukunaga et al., 2010^16^ | - Multicenter, prospective, open-label study (English) - Steroid refractory, moderate-to-severe UC | - LCAP | - Response | NR | NR | NR |
| Yoshimura et al., 2009^17^ | - RCT (English) - Moderate-to-severe UC | - GMA adjusted to body weight - GMA | - Remission | - SAE | NR | NR |
| Watanabe et al., 2016^18^ | - Prospective survey (English) | - Total proctocolectomy with IPAA | NR | NR | - IBDQ - Daily life satisfaction score | NR |

GMA, granulocyte and monocyte adsorption apheresis; IBDQ, inflammatory bowel disease questionnaire; IPAA, ileal pouch anal anastomosis; LCAP, leukocytapheresis; NR, not reported; PMS, post-marking surveillance; QoL, quality of life; RCT, randomized controlled trial; SAE, serious adverse event; UC, ulcerative colitis.

TABLE A5. Overview of included studies (Ichu-shi database).

| Authors, year | Study design (language) | Intervention | Efficacy | Safety | QoL | Mortality |  |
| --- | --- | --- | --- | --- | --- | --- | --- |
| Yoshimura et al., 2012^19^ | - Prospective, observational study (Japanese) - Grouped by steroid-dependent and steroid-refractory patients | - Infliximab | - Remission | - SAE requiring discontinuation - Flare requiring discontinuation - Flare | NR | NR |  |
| Nagayama et al., 2014^20^ | - Prospective, observational study (English) - Steroid-dependent and  steroid-refractory patients included | - LCAP | - Response - Remission | NR | NR | NR |  |
| Suzuki et al.,  2014^21^ | - Phase 2/3, double-blind RCT (English) | - Adalimumab - Placebo | - Response - Mucosal healing - Remission | - SAE requiring discontinuation - SAE - Infection - Flare - Malignancy | - IBDQ | NR |  |
| Kikuyama et al.,  2011^22^ | - Retrospective database study (English) - All patients are steroid refractory - Grouped by LBW, MBW, and HBW | - GMA | - Remission | NR | NR | NR |  |
| Yoshida et al.,  2011^23^ | - Prospective, single-arm, observational study (English) - Grouped by EU and NEU response group | - CAP | - Remission | NR | NR | NR | |
| Shimazu et al., 2016^24^ | - Prospective, single-arm, observational study (English) - Grouped by single- and double-needle GMA | - GMA | - Mucosal healing - Remission | NR | NR | NR | |
| Kobayashi et al., 2010^25^ | - Retrospective, medical record review study (English) | - Cyclosporin | - Response - Remission | - Surgery | NR | NR | |
| Hiwatashi et al., 2011^26^ | - Phase 3, double-blind RCT (English) - Moderate UC | - Mesalazine | - Response - Remission | - SAE | NR | NR | |
| Fukunaga et al.,  2006^27^ | - Prospective, single-arm observational study (English) - Severe UC | - LCAP | - Remission | NR | NR | NR | |
| Sawada et al.,  2016^28^ | - Retrospective, observational study (Japanese) - Steroid-dependent and steroid-refractory patients included | - CAP | - Response | NR | NR | NR | |
| Baba et al.,  2016^29^ | - Retrospective, observational study (Japanese) | - CAP | - Remission | NR | NR | NR | |
| Fukuchi et al., 2015^30^ | - Retrospective, observational study (Japanese) | - Mesalazine | - Response - Remission | NR | NR | NR | |
| Ubukata et al., 2014^31^ | - Prospective, single-arm, observational study (Japanese) | - Tacrolimus | - Mucosal healing - Remission | NR | NR | NR | |
| Yoshimura et al., 2014^32^ | - Prospective, observational study (Japanese) - All patients are steroid refractory | - Ciclosporin - Tacrolimus - Infliximab | - Remission | NR | NR | NR | |
| Ban et al., 2013^33^ | - Prospective, observational study (Japanese) | - Tacrolimus - Ciclosporin | - Response - Remission | NR | NR | NR | |
| Yoshimura et al.,  2013^34^ | - Prospective, observational study (Japanese) - All patients are steroid refractory | - Ciclosporin - Tacrolimus - Infliximab | - Remission | NR | NR | NR | |
| Ogiso et al., 2012^35^ | - Prospective, single-arm, observational study (Japanese) | - Mesalazine | - Remission | NR | NR | NR | |
| Shiga et al.,  2012^36^ | - Retrospective, observational study (Japanese) | - Mesalazine | - Remission | - SAE | NR | NR | |
| Takatsu et al., 2011^37^ | - Prospective, single-arm, observational study (Japanese) - Steroid-dependent and steroid-refractory patients included | - Tacrolimus | - Response - Remission | - SAE - Infection | NR | NR | |
| Kuriyama et al., 2011^38^ | - Retrospective, observational study (Japanese) - Steroid-dependent and steroid-refractory patients included | - Ciclosporin - Tacrolimus | - Response - Remission | NR | NR | NR | |
| Watanabe et al., 2005^39^ | - Prospective, observational study (Japanese) - Steroid-dependent and steroid-refractory patients included | - LCAP - GCAP | - Response - Remission | - Infection | NR | NR | |
| Ishikawa et al., 2004^40^ | - Prospective, observational study (Japanese) | - GCAP - GCAP + BDP - LCAP | - Remission | NR | NR | NR | |
| Miyata et al.,  2003^41^ | - Prospective, single-arm, observational study (Japanese) - Steroid-dependent and steroid-refractory patients included | - GCAP | - Response - Remission | NR | NR | NR | |

BDP, beclomethasone dipropionate; CAP, cytaphresis; EU, early ultrasonographic; GCAP, granulocytaoheresis; GMA, granulocyte and monocyte adsorption apheresis; HBW, high body weight; IBDQ, inflammatory bowel disease questionnaire; LBM, low body weight; LCAP, leukocytapheresis; MBW, medium body weight; NEU, non-early ultrasonographic; NR, not reported; RCT, randomized controlled trial; SAE, serious adverse event; UC, ulcerative colitis.

TABLE A6. Overview of included studies (additional handsearching).

| Authors, year | Study design (language) | Intervention | Efficacy | Safety | QoL | Mortality |
| --- | --- | --- | --- | --- | --- | --- |
| Motoya et al.,  2019^42^ | - Phase 3, double-blind RCT (English) - Grouped by steroid-dependent and steroid-refractory patients | - Vedolizumab - Placebo | - Response - Remission - Mucosal healing | - SAE - SAE requiring discontinuation | NR | Reported |

## RCT, randomized controlled trial; NR, not reported; QoL, quality of life; SAE, serious adverse event.
